# Employment of Glycerine in the Treatment of Diseases of the Eye

**Published:** 1865-02

**Authors:** W. Abbotts Smith


### *Employment of Glycerine in the Treatment of Diseases of the Eye.* By W. Abbotts Smith, M. D.

Notwithstanding that glycerine is largely used as a bland, emolient application to the skin, in several affections of that delicate membrane, its importance as a remedial agent in certain diseases of the conjunctiva, appears to be considerably underrated, although several marked points of analogy exist between the two structures.

Having been induced some time since by the perusal of a paper published in the *Bulletin de Therapeutique*, by M. Foucher, upon this subject, I have given a fair trial to the topical employment of glycerine, and the results have been such as to warrant me in recommending its use in some of the forms of opthalmia, and other superficial affections of the eye.

The application of *pure* glycerine, uncombined, is decidedly advantageous for the prevention and removal of the hard crusts produced by irritation which are occasionally closely adherent to the eye-lids in ophthalmia, which disorder they consequently tend to perpetuate.

In ectropion, and in cases of partial loss of the eye-lashes, glycerine forms an agreeable and efficient application; and it will also be found similarly useful in epiphora, and in any other superficial diseases of the eye which are dependent upon diminished secretion by the Meibomian glands Among these may be especial mentioned the peculiar condition of the conjunctiva to which Von Ammon gave the name of Xerosis Conjunctivæ, in reference to the dry, cuticular appearance of the eye-ball, and which is most commonly met with in persons of scrofulous diathesis, who have long suffered from chronic ophthalmia. The free play of the eye-lids upon the front surface of the eye-ball is preserved by the topical employment of the glycerine; and the tendency to symblepharon, or to an uncomfortable temporary adhesion of the eyelids, which occurs after sleep, is counteracted.

Unctuous applications are always preferable to watery solutions in ophthalmia, both because they are not so readily washed away by the tears, and because they more closely resemble the natural greasy secretion of the Meibomian glands, by which the conjunctival surface is lubricated in the healthy state; and the circumstance that glycerine alone, when employed locally, is highly beneficial in the ophthalmic affections already referred to, also points to that substance as a valuable vehicle in the preparation of collyria.

M. Foucher enumerates, in his paper in the *Bulletin de Therapeutique*, a long list of medicaments with which he has combined glycerine in the treatment of ophthalmia in numerous cases which had come under his care at the Hopital de Enfants. The substances which I have found to be most usefully employed in this form are the sulphate of zinc, the sulphate of copper, and laudanum; but M. Foucher also combines glycerine with the perchloride of iron, with borax, with tincture of iodine, and other agents.

It should be here stated that the nitrate of silver, owing to the chemical action which ensues upon its coming in contact with organic matter, cannot be used conjointly with glycerine; and it further remains to be mentioned that the glycerine should be well purified, in which condition it is perfectly natural, does not possess any odor or taste, and does not produce irritation when it is applied to the eye.

